# Alzheimer’s disease master regulators analysis: search for potential molecular targets and drug repositioning candidates

**DOI:** 10.1186/s13195-018-0394-7

**Published:** 2018-06-23

**Authors:** D. M. Vargas, M. A. De Bastiani, E. R. Zimmer, F. Klamt

**Affiliations:** 1 Laboratory of Cellular Biochemistry, Biochemistry Department, Institute of Health Sciences (ICBS), Federal University of Rio Grande do Sul (UFRGS), Porto Alegre, RS 90035-003 Brazil; 2 Pharmacology Department, Institute of Health Sciences (ICBS), Federal University of Rio Grande do Sul (UFRGS), Porto Alegre, RS 90035-003 Brazil; 3 Brain Institute of Rio Grande do Sul (BraIns), Pontifical Catholic University of Rio Grande do Sul (PUCRS), Porto Alegre, RS 90619-900 Brazil; 4 National Science Technology Institute for Translational Medicine (INCT-TM), National Council for Scientific and Technological Development (CNPq), Porto Alegre, Brazil

**Keywords:** Alzheimer’s disease, Hippocampus, Transcriptional regulatory network reconstruction, Master regulators, Drug repositioning, Transcription factors

## Abstract

**Background:**

Alzheimer’s disease (AD) is a multifactorial and complex neuropathology that involves impairment of many intricate molecular mechanisms. Despite recent advances, AD pathophysiological characterization remains incomplete, which hampers the development of effective treatments. In fact, currently, there are no effective pharmacological treatments for AD. Integrative strategies such as transcription regulatory network and master regulator analyses exemplify promising new approaches to study complex diseases and may help in the identification of potential pharmacological targets.

**Methods:**

In this study, we used transcription regulatory network and master regulator analyses on transcriptomic data of human hippocampus to identify transcription factors (TFs) that can potentially act as master regulators in AD. All expression profiles were obtained from the Gene Expression Omnibus database using the GEOquery package. A normal hippocampus transcription factor-centered regulatory network was reconstructed using the ARACNe algorithm. Master regulator analysis and two-tail gene set enrichment analysis were employed to evaluate the inferred regulatory units in AD case-control studies. Finally, we used a connectivity map adaptation to prospect new potential therapeutic interventions by drug repurposing.

**Results:**

We identified TFs with already reported involvement in AD, such as ATF2 and PARK2, as well as possible new targets for future investigations, such as CNOT7, CSRNP2, SLC30A9, and TSC22D1. Furthermore, Connectivity Map Analysis adaptation suggested the repositioning of six FDA-approved drugs that can potentially modulate master regulator candidate regulatory units (Cefuroxime, Cyproterone, Dydrogesterone, Metrizamide, Trimethadione, and Vorinostat).

**Conclusions:**

Using a transcription factor-centered regulatory network reconstruction we were able to identify several potential molecular targets and six drug candidates for repositioning in AD. Our study provides further support for the use of bioinformatics tools as exploratory strategies in neurodegenerative diseases research, and also provides new perspectives on molecular targets and drug therapies for future investigation and validation in AD.

**Electronic supplementary material:**

The online version of this article (10.1186/s13195-018-0394-7) contains supplementary material, which is available to authorized users.

## Background

Alzheimer’s disease (AD) is the most prevalent neurodegenerative disease and the major cause of dementia. In the United States about 10% of people over the age of 65 years have Alzheimer’s dementia, and the worldwide prevalence of the disease ranges from 4 to 8% [1, 2]. A total of roughly 46 million AD cases is estimated around the world and related cost are about U$800 billion per year [2, 3].

This neurodegenerative disease causes gradual loss of brain volume and synaptic dysfunction, leading to a progressive memory and reasoning impairment followed by global cognitive decline and, ultimately, dementia [4, 5]. AD is characterized by its histopathological hallmarks, which includes deposits of amyloid-β (Aβ) plaques and neurofibrillary tangles composed of hyperphosphorylated tau [6]. Recent advances in our ability to detect AD pathophysiology using imaging biomarkers currently allow the identification of Aβ and tau pathology in living individuals [7, 8]. By contrast, few advancements have been made in terms of drug treatments, which currently are available only for ameliorating symptoms [9].

Sporadic AD, also called late-onset AD, represents the vast majority of cases (> 95%) and is recognized as a multifactorial, complex disease [6]. Apolipoprotein E isoform ε4 (APOEε4) is the main susceptibility gene for AD, with a threefold increase in AD risk for one allele and 12-fold increase for two alleles [10]. Genome-wide association studies have identified more than 20 AD risk genes and several disease-associated pathways [11]. However, the AD risk genes identified so far are neither necessary nor sufficient for disease onset [12]. Meanwhile, evidence suggest that nongenetic factors, such as cerebrovascular disease, diabetes, and obesity, also increase the risk of developing AD [6]. Furthermore, gene expression profiling studies in AD brains have shown many genes working together in relevant altered biological pathways in the disease, leading to a growing acceptance that AD results from the impairment of several complex mechanisms at once that have not yet been fully elucidated [13].

In keeping with this, the high rate of failure in the development of AD-modifying therapies seems to be a consequence of the incomplete knowledge about the underlying mechanisms of the disease. Based on this, the use of new approaches to study the disease pathophysiology and search for alternative therapeutic targets are urgently required [9, 14, 15]. The use of integrative strategies, such as regulatory networks, for analyzing high-throughput expression data have produced significant knowledge towards the elucidation of biological mechanisms underlying complex diseases, such as cancer and obesity [16]. Furthermore, it has been observed that regulatory networks inferred by reverse engineering algorithms can provide sufficient accuracy to estimate the impact of transcription factors (TFs) on phenotype transitions according to their transcriptional targets, and to identify the ones that are acting as master regulators (MRs) of diseases [17]. Many approaches have shown that TFs can operate as key elements in the phenotypic determination by regulating large groups of transcriptional targets associated with complex cellular processes [17–20]. Therefore, the analysis of expression profiling data using a TF-centered regulatory networks approach seems an interesting strategy to study the mechanisms and common drivers associated with AD.

In this study, gene expression data available in the Gene Expression Omnibus repository (GEO; http://www.ncbi.nlm.nih.gov/geo/) was used to infer a transcriptional regulatory network, through reverse engineering, for the human hippocampus, a region that undergoes high rates of volume loss in AD. Afterwards, expression data from AD case-control studies of the same region were used to identify MRs potentially modulating phenotypic changes from a normal to a pathological scenario. Moreover, the prospection of new drug candidates to treat AD patients was carried out by a connectivity map approach using the inferred regulatory units of MR candidates.

## Methods

### Microarray data acquisition

A normal human brain expression dataset was obtained from the GEO database under the accession number GSE60862 [21]. AD case-control microarray studies from hippocampal samples were acquired from GEO under accession numbers GSE5281 [22, 23], GSE29378 [24], GSE36980 [25], and GSE48350 [26]. Table 1 summarizes the data information from the selected GEO datasets used in this study. Each expression dataset was treated and analyzed independently (Additional file 1: Figure S1).

**Table 1:** Gene expression microarray data used to infer human hippocampus transcriptional network and AD MR candidates

GEO ID	Description	Samples (*n*)	Reference
GSE60862	Gene expression data of 10 regions of postmortem brains originating from 134 neurologically and neuropathologically normal Caucasian individuals	Hippocampus (*n* = 114)	Trabzuni et al., 2011 [21]
GSE5281	Gene expression data of 6 regions of postmortem brains originating from 33 Alzheimer’s disease and 14 neurologically normal aged individuals	Hippocampus AD individuals (*n* = 10) Hippocampus normal individuals (*n* = 13)	Liang et al., 2007 [22]; Liang et al., 2008 [23]
GSE29378	Gene expression data of the CA1 and CA3 hippocampus regions of postmortem brains from 17 Alzheimer’s disease and 16 neurologically normal aged individuals	Hippocampus AD individuals (CA1 *n* = 16, CA3 *n* = 15) Hippocampus normal individuals (CA1 *n* = 16, CA3 *n* = 16)	Miller et al., 2013 [24]
GSE36980	Gene expression data of frontal and temporal cortices and hippocampal regions of postmortem brains originating from 26 Alzheimer’s disease and 62 neurologically normal aged individuals	Hippocampus AD individuals (*n* = 7) Hippocampus normal individuals (*n* = 10)	Hokama et al., 2014 [25]
GSE48350	Gene expression data of 4 regions of postmortem brains originating from 26 Alzheimer’s disease and 33 neurologically normal aged individuals	Hippocampus AD individuals (*n* = 17) Hippocampus normal individuals (*n* = 23)	Berchtold et al., 2013 [26]

*AD* Alzhimer’s disease, *MR* master regulator

### Region-specific transcription network inference

The genome-wide region-specific transcriptional network (TN) centered on TFs and their predicted target genes were inferred using the normal brain hippocampus (HIP) expression data from GSE60862. The groups of inferred target genes associated with each TF are hereinafter referred as its regulatory unit. These computations were performed using the RTN package, which is designed to reconstruct and analyze TNs based on the mutual information (MI), a measure that evaluates dependencies between two random variables, using the ARACNe (Algorithm for the Reconstruction of Accurate Cellular Networks) method. Briefly, the regulatory structure of the network is derived by mapping significant associations between known TFs and all potential targets. Interactions below a minimum MI threshold are eliminated by a permutation step and unstable interactions are additionally removed by bootstrap to create a consensus bootstrap network. In a final step, the data processing inequality algorithm is applied with null tolerance to eliminate interactions that are likely to be mediated by another TF. Here, we used the package’s default number of permutations and number of bootstraps (1000 permutations and 100 bootstraps), but with a *p* value cutoff of 0.001. The resultant network will be hereinafter referred to as HIP-TN [18, 27, 28].

All computational analyses were performed in R statistical environment [29]. Network figures were constructed with the RedeR graphical platform for exploration of biological networks [30], and other plots were constructed using ggplot2 [31].

### Master regulators and gene set enrichment analysis

After the hippocampus transcriptional regulatory network (HIP-TN) inference, we applied the master regulator analysis (MRA) algorithm described by Carro et al. [17] to the regulatory units comprised of at least 100 targets. The algorithm computes the statistical significance of the overlap between the regulatory units in HIP-TN and the differentially expressed genes (false discovery rate (FDR)-adjusted *p* value < 0.05) obtained from each AD study, corrected for multiple comparisons. We then selected the regulatory units of the TFs showing significant enrichment of differentially expressed target genes in three or more studies, which we termed MR candidates.

Two-tail gene set enrichment analysis (GSEA) was also performed using the RTN package with 1000 permutations, as previously described [32]. Briefly, the groups of target genes for each MR (regulatory units) were split into positive and negative mode of action targets using Pearson’s correlation. Next, the association of each subgroup was assessed by GSEA statistics in each ranked phenotype, resulting in independent enrichment scores (Es), with two enrichment distributions. Additionally, a differential enrichment was performed among subgroups (EsA-EsB) where maximum deviation from zero near opposite extremes is desirable for a clear association. Thus, a highly positive differential score implies that the regulatory unit is induced in the disease phenotype, while a highly negative differential score indicates that the regulatory unit is repressed in the disease phenotype. The two-tail GSEA computation *p* value cutoff was set to 0.05 and 1000 permutations were used.

The differentially expressed genes used in the MRA and the log fold change (logFC) metric used to obtain the ranked phenotypes required for the GSEA were computed using the Bioconductor package limma [33].

### Connectivity map drug profiling approach

The previously identified MR candidate regulatory units were queried in the Connectivity Map online tool (The CMap build02; www.broadinstitute.org/cmap/) using the GSEA algorithm described by Lamb et al. [34]. This tool compares queried signature with gene expression profile database of several cell lines after treatment with approximately 1000 compounds, most of which are FDA approved. Drugs whose signature opposes the disease signature are assumed to have a therapeutic potential.

For this, we first selected the MR candidates with two-tail GSEA *p* values less or equal to 0.01. Next, for each case-control study, the differentially expressed targets of these MR candidates (adjusted *p* value < 0.05) were filtered, grouped, tagged according to the logFC metric, converted to Affymetrix probe identifiers, and submitted as input for the cMap webtool. Then we obtained a connectivity map of drug-phenotype association for each case-control study.

## Results

### Human hippocampus transcriptional regulatory network reconstruction

**Fig. 1:**
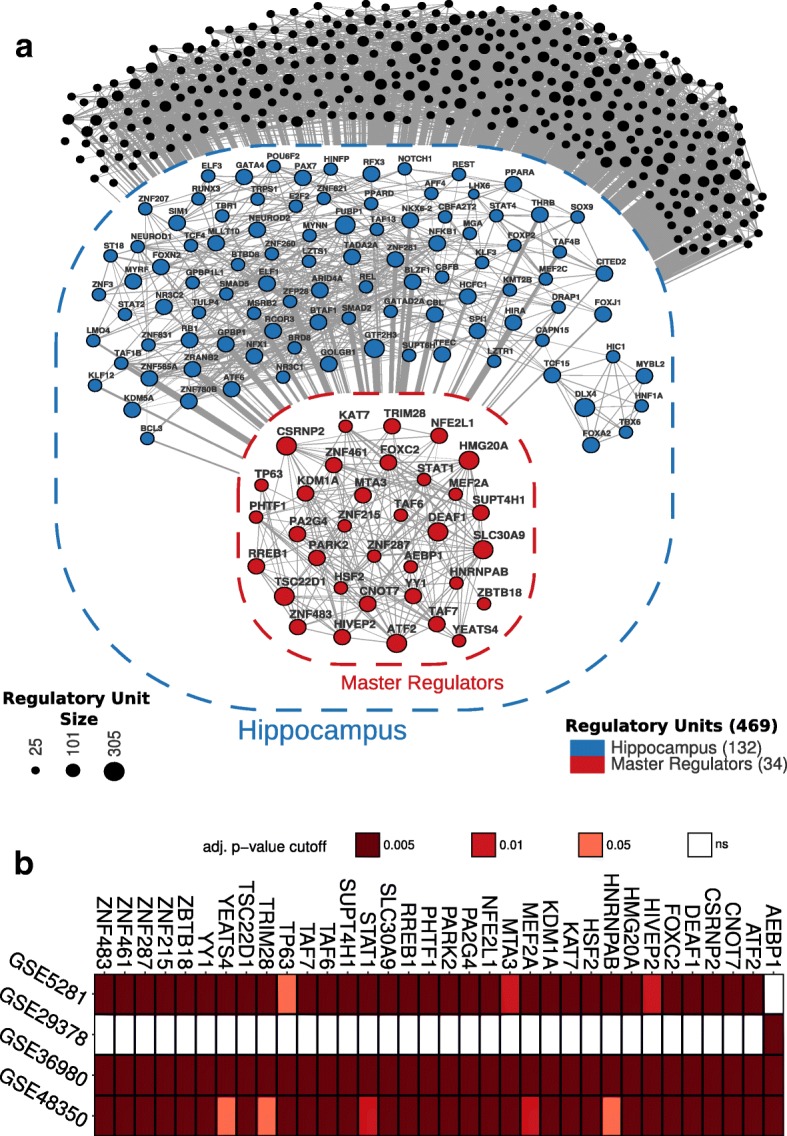
Transcriptional regulatory network and master regulators. **a** Human hippocampus transcription regulatory network centered on transcription factors was reconstructed from normal brain dataset (GSE60862). The network shows 469 regulatory units of transcripts classified under transcription factor activity, sequence-specific DNA binding (GO:0003714), with more than 25 inferred targets; 132 of them showed more than 100 inferred targets and were considered tissue-specific regulatory units (blue container). These were then tested in AD case-control studies using master regulator analysis, resulting in 34 tissue-specific regulatory units significantly enriched with differentially expressed genes (red container). The 337 regulatory units with less than 100 targets are represented in black outside the blue container. **b** Tile plot representation of the MR candidates for each case-control expression dataset (GSE5281, GSE29378, GSE36980, and GSE48350). ns not significant

HIP-TN was computed from a normal brain gene expression dataset (GSE60862) using the reverse engineering ARACNe algorithm. Transcripts were classified as transcription factors when annotated in the Gene Ontology with the identifier GO:0003700 (transcription factor activity, sequence-specific DNA binding). Among a total of 20,311 transcripts in the dataset, 766 were annotated as TFs under GO:0003700. From these, 469 were classified as TFs with more than 25 inferred target genes. The resultant HIP-TN, comprising 132 regulatory units with more than 100 targets, was used for further analyses. Figure 1a shows the inferred HIP-TN inside the blue container, where each node symbolizes a TF regulatory unit and node sizes correspond to the number of predicted targets for each TF (Additional file 2: Table S1). Regulatory units with less than 100 targets are represented in black outside the blue container.

### Hippocampus AD master regulator inference

Microarray gene expression from AD case-control studies available in GEO (GSE5281, GSE29378, GSE36980, and GSE48350) were used to obtain disease MR candidates considering the normal HIP-TN previously inferred. MRA was performed to evaluate HIP regulatory units enriched with genes differentially expressed between the two phenotypes (disease and control). Only regulatory units significantly enriched in at least three case-control studies were considered as MR candidates. These analyses resulted in the identification of 34 MR candidates (Fig. 1b) (Additional file 3: Table S2).

**Fig. 2:**
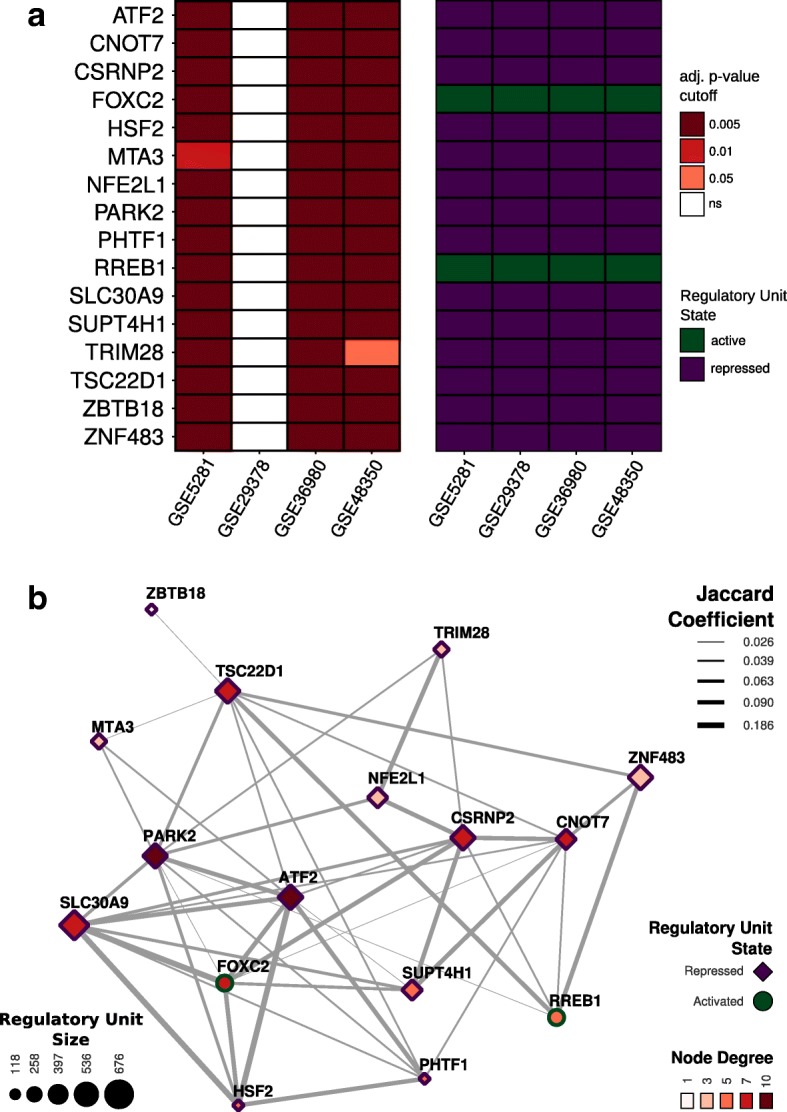
Activation state of MR candidates and AD subregulatory network. **a** Tile plot representing the state of activation of MR candidates (two-tail gene set enrichment analysis) for each case-control expression dataset. **b** Subregulatory network showing the associations between the significantly activated and repressed MR candidates. Node size represents the number of inferred targets of the master regulator transcription factor candidate; node shape shows their activation state; node color maps their connectivity (subnetwork average degree = 5.75 ± 2.65); edge width shows the Jaccard coefficient of common targets between transcription factor pairs. ns not significant

Two-tail GSEA was performed to infer the activation state of each MR candidate. The outcome of this analysis showed 14 MR candidates that were significantly repressed and 2 MR candidates that were significantly activated in AD (FDR adjusted *p* value ≤ 0.05) (Fig. 2a). This means that targets from the repressed MR candidates had predicted positive TF-target expression association under normal conditions but were decreased in the disease. To the contrary, targets with inferred negative TF-target association under normal conditions had increased expression in the pathology. For the activated MR candidates, the inferred positive or negative TF target expression associations do not reverse during AD. The remaining 18 MR candidates did not present statistically significant results regarding their activation states and thus were not considered for the next steps.

The AD subregulatory network graph in Fig. 2b and Additional file 4 (Table S3) shows the association pattern between those MR candidates with significant alteration of the activation state in the disease state compared with control. The nodes with the highest degrees of connectivity in this network correspond to the MRs ATF2 (activating transcription factor 2) and PARK2 (Parkin RBR E3 ubiquitin protein ligase). The number of common targets between any two MRs are represented by the connector line widths as assessed by the Jaccard coefficient and indicates that certain variations in target expression may be a result of the contiguous regulatory action of two or more TFs.

### Connectivity map

**Fig. 3:**
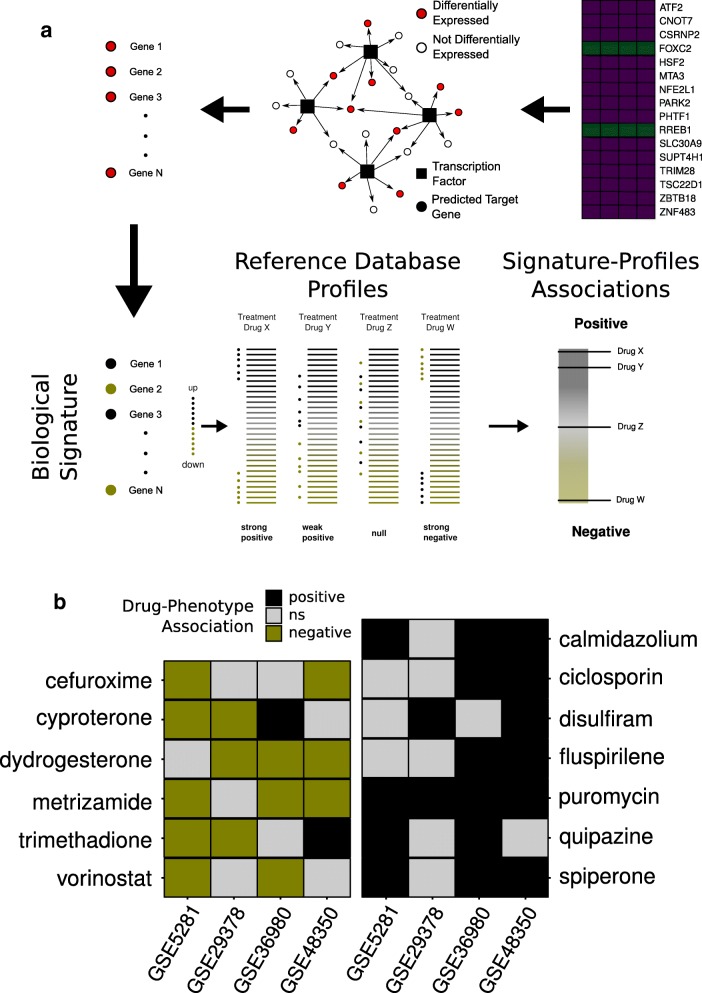
Connectivity map analysis and drug repurposing to AD therapy. **a** Schematic representation of connectivity map analysis: differentially expressed targets of repressed or activated MR candidates, for each case-control study, were ranked and used as query signature to the connectivity map webtool against gene expression profiles database of several cell lines treated with thousands of FDA-approved compounds. **b** Case-control associated drugs: consensus drugs consistently matched with at least two case-control studies. Drugs with negative AD association are assumed with therapeutic potential, and the ones with positive association are considered AD mimetic. ns not significant

The connectivity map approach was used to search for drugs with therapeutic repurposing potential in AD. The 16 MR candidate regulatory units identified in the previous analysis were grouped, and their up- or downregulated differentially expressed targets were selected for each AD case-control studies and used as an input in the webtool (Fig. 3a). The consensus drugs are consistently present in at least two case-control studies (*p* ≤ 0.05). Six drugs were negatively associated with AD and assumed to have a therapeutic potential: Cefuroxime, Cyproterone, Dydrogesterone, Metrizamide, Trimethadione, and Vorinostat. Additionally, seven drugs were positively associated and thus considered AD mimetic: Calmidazolium, Ciclosporin, Disulfiram, Fluspirilene, Puromycin, Quipazine, and Spiperone (Fig. 3b) (Additional file 5: Table S4).

## Discussion

Based on reverse engineering coexpression regulatory network reconstruction for the human HIP, we identified a range of transcription factors that acts on large regulatory units, therefore being potentially important for the functionality of this region. Furthermore, from these regulatory units, we selected those which differentially expressed inferred target genes were overrepresented in AD versus control. Interestingly, among the ten largest hippocampal regulatory units, seven of them present differential expression when comparing AD versus control (Additional file 2: Table S1).

The 34 MR candidates selected in this study were annotated with the GO term “transcription factor activity, sequence-specific DNA binding”. Among them, only 5 MR candidates, namely KAT7, MTA3, RREB1, TSC22D1, and ZNF287, do not have this GO term assigned by a curator. Moreover, some of the MR candidates have also been associated with other transcription regulation functions, such as transcription corepressor activity (GO:0003714) and transcription coactivator activity (GO:0003713), bringing forth the possibility that the influence of each MR candidate on the expression levels of its inferred regulatory units may be related to expression regulation mechanisms other than the direct DNA binding activity. Nevertheless, it is important to note that, for the purpose of finding MRs for the disease, this distinction is not necessarily relevant once the sought MR function relates to the expression regulation as a broad and diverse regulatory phenomenon. The additional GO terms annotated for each MR candidate are described in Additional file 3 (Table S2).

Among the TFs inferred as MRs of the disease, several, such as ATF2 and PARK2, have already had their relationship with AD previously reported [35–37]. Indeed, both *ATF2* and *PARK2* showed a high degree of connectivity via their inferred targets in the AD regulatory subnetwork, thus acting as potential hubs, and predicted elements of great importance for network maintenance and robustness, with their regulatory units repressed in the disease.

The *PARK2* gene encodes an E3 ubiquitin ligase and it is one of the genes involved in autosomal recessive juvenile parkinsonism [38]. In addition to its function in the ubiquitin proteasome system, PARK2 is also involved in the regulation of gene expression, modulating genes associated with apoptosis or cellular stress reactions [39]. Furthermore, PARK2 also acts as a direct transcriptional repressor of p53 promotor activity, thus modulating cell death pathways [40]. Remarkably, PARK2 was also shown to directly mediate expression of two proteins related to the amyloidogenic pathway, Presenilin 1 and Presenilin 2, which are components of the γ-secretase complex [35]. Mutations in the coding regions of these two proteins are related to AD familial cases [41].

ATF2 is a member of the ATF/CREB family that regulates gene expression through homodimerization or heterodimerization with several other protein partners. However, the role of each dimer in target regulation is very difficult to determine and the knowledge about them is still limited [42]. ATF2 is activated by several cell-damaging stimuli, such as cisplatin-induced genotoxic stress and ultraviolet (UV) radiation exposure [43, 44]. This TF regulates the expression of genes involved in important cellular processes also altered in AD, such as inflammatory signaling, apoptotic pathway, DNA damage response, and cell cycling control, being regarded as an early stress response protein [42, 45–48]. In agreement with the results obtained in this study, reduced expression of ATF2 has been shown for the CA1 to CA4 hippocampal areas, granule cells of the dentate gyrus, and adjacent entorhinal cortex in AD patients [36, 37].

Furthermore, nuclear availability of ATF2 and PARK2 are strongly influenced by stressing factors. ATF2 translocation from the nucleus to the cytoplasm was found to be increased in situations of cellular stress and disease states, leading to cell death triggering by induced opening of mitochondrial membrane pores [46]. PARK2 solubility is compromised by oxidative and nitrosative stress and aging, in some cases showing behavioral patterns equivalent to those *PARK2* mutations correlated with Parkinson’s disease [38]. Thus, the reduced nuclear availabilities of both TFs in response to severe stress may account for the target expression reductions identified in this study.

We also identified novel TFs that seem to be involved in AD: CNOT7 (CCR4-NOT transcription complex subunit 7), CSRNP2 (cysteine and serine rich nuclear protein 2), SLC30A9 (solute carrier family 30 member 9), and TSC22D1 (TSC22 domain family member 1). These MR candidates have also shown a high degree of connectivity in the AD subregulatory network, being also potentially important for this disease. In the following sections we discuss each of these MRs with a brief description of its known functions.

CNOT7 is a catalytic component of one of the major mRNA deadenylase complexes (CCR4-NOT). It has an antiproliferative function dependent both on its deadenylase activity and its association with BTG1 (BTG anti-proliferation factor 1) [49, 50]. SLC30A9, also called ZnT9, belongs to a family of zinc transporters. This protein contains a motif for interaction with nuclear receptors, apparently migrating to the nucleus in a cell cycle-dependent manner [51, 52]. It has been shown that SLC30A9 acts as a hormone-dependent nuclear receptor coactivator and also participates in the Wnt signaling pathway by interacting with β-catenin [53, 54]. TSC22D1 is the most studied among these transcription factors due to its tumor suppressor activity. It was isolated as a transforming growth factor (TGF)-β-induced transcript which encodes a leucine-zipper transcription factor and has transcriptional repressor activity [55, 56]. TSC22D1 has been shown to be a p53-positive regulator, inhibiting its degradation. Furthermore, it also inhibits cell proliferation, promoting apoptosis when overexpressed [57].

Although it was not possible to determine the activation state of several other MRs found, a handful of studies directly correlating MEF2A (myocyte enhancer factor 2A), STAT1 (signal transducer and activator of transcription 1), and YY1 (Yin and Yang 1 protein) TFs to AD are available in the literature. It was shown that YY1 is directly involved in the regulation of important AD-related genes, such as *BACE1* (Beta-secretase 1) and *APH1A* (aph-1 homolog A, gamma-secretase subunit), which have binding sites for YY1 in their promoter regions [58–60]. STAT1 also has a role in controlling the gene expression of *BACE1*, binding to its promoter region, and can be upregulated by Aβ, characterizing a positive feedback loop that could lead to the progressive increase of production and further accumulation of Aβ [61–63]. Regarding MEF2A, Burton et al. [64] and Gonzalez et al. [65] have suggested that deregulation in the control of these TF activation pathways could be associated with increased risk of developing AD. Additionally, the genes MEF2C and CELF1, identified by genome-wide association studies as having a small effect on AD risk [11], were inferred as MEF2A and YY1 targets, respectively (data not shown), which reinforces the idea that these genes are part of a broad and complex context and that to discuss their roles in the whole scenario could be a much more constructive approach.

Neuronal loss and astrocytosis are well-known events related to AD, and both have been observed in postmortem brains of AD patients [66–68]. A reduction in the neuronal population is directly related to the progression of hippocampal atrophy, to the severity of the dementia [69, 70], and to the Braak stage of the disease [71]. The presence of astrocytosis in AD has also been described, and it is thought to be related either to the proliferation of astrocytes to replace dying neurons, or to an increased activity of these cells in an effort to scavenge the toxic Aβ peptides [72, 73]. Although astrocytosis is known to be essential for tissue repair and early mitigation of lesions, it can also lead to further deleterious effects, either by amplifying the inflammatory response [73] or by diminishing the trophic support for neurons [74, 75].

To investigate whether our results could be related to these histopathological alterations, we conducted a preliminary GSEA to compare the expression levels of the 34 regulatory units (HIP-TN) in mouse neuron versus astrocyte data from the microarray dataset GSE9566 [76]. We found that all 34 regulatory units were enriched with differentially expressed genes in astrocytes compared with neurons. Notably, 16 regulatory units followed the same pattern of activation found in the AD case-control analysis, whereas 18 showed nonsignificant states of activation (Additional file 6: Figure S2). These findings indicate that our results can be, at least in part, a reflex to an increase in the influence of astrocyte-related regulatory units in the overall signature of the disease, which may be compatible with the astrocyte hyperactivation and proliferation hypothesis in AD. Therefore, a reversion of the inferred transcriptional signature as a whole can be a promising strategy to alleviate deleterious effects potentially mediated by these responses.

The transition from a single-gene approach to a network-centric view is seen as a new path in the search for pharmacological strategies for complex diseases [77]. In addition, drug repositioning has been shown to be a cheaper and faster alternative method for the development of new therapeutic regimens [14]. The connectivity map proposal enables us to combine both of these paradigms by incorporating a data-driven method for exploring transcriptional profile alterations with drug effects on expression. We applied a connectivity map adaptation centered on transcription factor regulatory units and obtained six FDA-approved drug candidates that seem to revert AD phenotype (Cefuroxime, Cyproterone, Dydrogesterone, Metrizamide, Trimethadione, and Vorinostat). Interestingly, these drugs have several self-related or class-related neuroprotective effects previously reported in the literature. Notably, Cyproterone and Vorinostat have already been shown to be neuroprotective in AD models. Cyproterone is an antiandrogen that antagonizes androgen-mediated gene expression, although it exerts a testosterone-like neuroprotective effect against Aβ toxicity in primary neuronal cultures by an androgen receptor activation-dependent mechanism [78]. Vorinostat is a histone deacetylase inhibitor (HDACi) used for cancer treatment, and it has been shown to restore memory deficits in an AD animal model and protects against Aβ toxicity in an AD cell model [79]. This drug is currently at phase 1 clinical trial for assessment of its memory performance improvement capabilities in AD [80] (www.clinicaltrial.gov). Furthermore, there are several studies showing the role of HDACis in the reduction of inflammatory mediator expression, excitotoxicity, and oxidative stress, as well as enhancement of neurotrophic factor expression, which are relevant pathways for AD [81]. Trimethadione is a T-type calcium channel inhibitor used as an anticonvulsant drug. It has been reported as a neuroprotective compound leading to both prevention of calcium homeostasis impairment, potentially associated with the onset of AD, and reduction of age-related degenerative effects in animal models [82, 83].

Cefuroxime is a second-generation cephalosporin antibiotic that can cross the blood-brain barrier, and Dydrogesterone is a progestogen usually administered in conditions associated with progesterone deficiency [84, 85]. Although neither of them has reported neuroprotective effects, there are several class-related central nervous system benefits associated with them in the literature [86–88]. Finally, Metrizamide, a radiocontrast shown to effectively inhibit the brain hexokinase, has a recent pharmacodynamic study exploring its effects on neuronal function [89].

## Conclusion

Systems biology is an integrative, hypothesis-free approach based on biological component interactions and represents an interesting avenue to study complex diseases. Indeed, regulatory networks centered in TF have already been shown effective in identifying cancer drivers [17, 18, 32]. Furthermore, this approach is also gradually becoming the methodology of choice to study multifactorial complex neurodegenerative diseases [19, 90, 91]. Herein, employing a systems approach, we identified several TFs previously related to the disease as well as novel potential targets to be investigated. In addition, new therapeutic strategies using drug repositioning were prospected from the obtained transcriptional signatures. Nevertheless, further studies using both in vitro and in vivo models are required to fully evaluate the impact and benefits of these findings in AD.

## Additional files

Additional file 1: **Figure S1.** Methodology flowchart. (A) Publicly available expression profile from Gene Expression Omnibus (GEO) were retrieved for normal brain hippocampus (GSE60862) and AD case versus control hippocampus (GSE5281, GSE29378, GSE36980, GSE48350). (B) Normal brain dataset was submitted to reverse engineering TF-centered transcription network reconstruction using ARACNe algorithm. Inferred healthy hippocampus regulatory units were then employed to query the master regulators of AD using master regulator analysis. Finally, the master regulator candidates were investigated for their state of activation using two-tail GSEA and possible repurposing drugs using connectivity maps. (PDF 5913 kb)

Additional file 2: **Table S1.** Hippocampus transcription factor-centered network nodes and edges information. (XLSX 40 kb)

Additional file 3: **Table S2.** Master regulators analysis results. (XLSX 23 kb)

Additional file 4: **Table S3.** Master regulator candidates subnetwork nodes and edges information. (XLSX 12 kb)

Additional file 5: **Table S4.** Connectivity map of master regulator candidates. (XLSX 11 kb)

Additional file 6: **Figure S2.** Activation state of MR candidates in AD case-control studies and mouse neuron versus astrocyte data. Tile plot representing the MR candidate state of activation (two-tail gene set enrichment analysis) for the AD case-control (GSE5281, GSE29378, GSE36980, and GSE48350) and mouse neuron versus astrocyte (GSE9566) expression datasets. (PDF 24 kb)

## Abbreviations

AD: Alzheimer’s disease
APH1A: APH-1 homolog A, gamma secretase subunit
APOEε4: Apolipoprotein E isoform ε4
ARACNe: Algorithm for the Reconstruction of Accurate Cellular Networks
ATF2: Activating transcription factor 2
Aβ: Amyloid-β
BACE1: Beta-secretase 1
CNOT7: CCR4-NOT transcription complex subunit 7
CSRNP2: Cysteine and serine rich nuclear protein 2
Es: Enrichment scores
FDR: False discovery rate
GEO: Gene Expression Omnibus
GSEA: Gene set enrichment analysis
HDACi: Histone deacetylase inhibitor
HIP: Hippocampus
HIP-TN: Hippocampus transcriptional network
LogFC: Log fold change
MEF2A: Myocyte enhancer factor 2A
MI: Mutual information
MR: Master regulator
MRA: Master regulator analysis
PARK2: Parkin RBR E3 ubiquitin protein ligase
SLC30A9: Solute carrier family 30 member 9
STAT1: Signal transducer and activator of transcription 1
TF: Transcription factor
TN: Transcriptional network
TSC22D1: TSC22 domain family member 1
YY1: Yin and Yang 1 protein
