# Supplementary material for: Alzheimer’s disease master regulators analysis: search for potential molecular targets and drug repositioning candidates
Source: Alzheimers Res Ther. 2018 Jun 23;10:59. doi: 10.1186/s13195-018-0394-7 (PMC6015462; doi:10.1186/s13195-018-0394-7)
Supplement: Supplementary file 1 — Figure S1. Methodology flowchart. (A) Publicly available expression profile from Gene Expression Omnibus (GEO) were retrieved for normal brain hippocampus (GSE60862) and AD case versus control hippocampus (GSE5281, GSE29378, GSE36980, GSE48350). (B) Normal brain dataset was submitted to reverse engineering TF-centered transcription network reconstruction using ARACNe algorithm. Inferred healthy hippocampus regulatory units were then employed to query the master regulators of AD using master regulator analysis. Finally, the master regulator candidates were investigated for their state of activation using two-tail GSEA and possible repurposing drugs using connectivity maps. (PDF 5913 kb) [file 13195_2018_394_MOESM1_ESM.pdf]

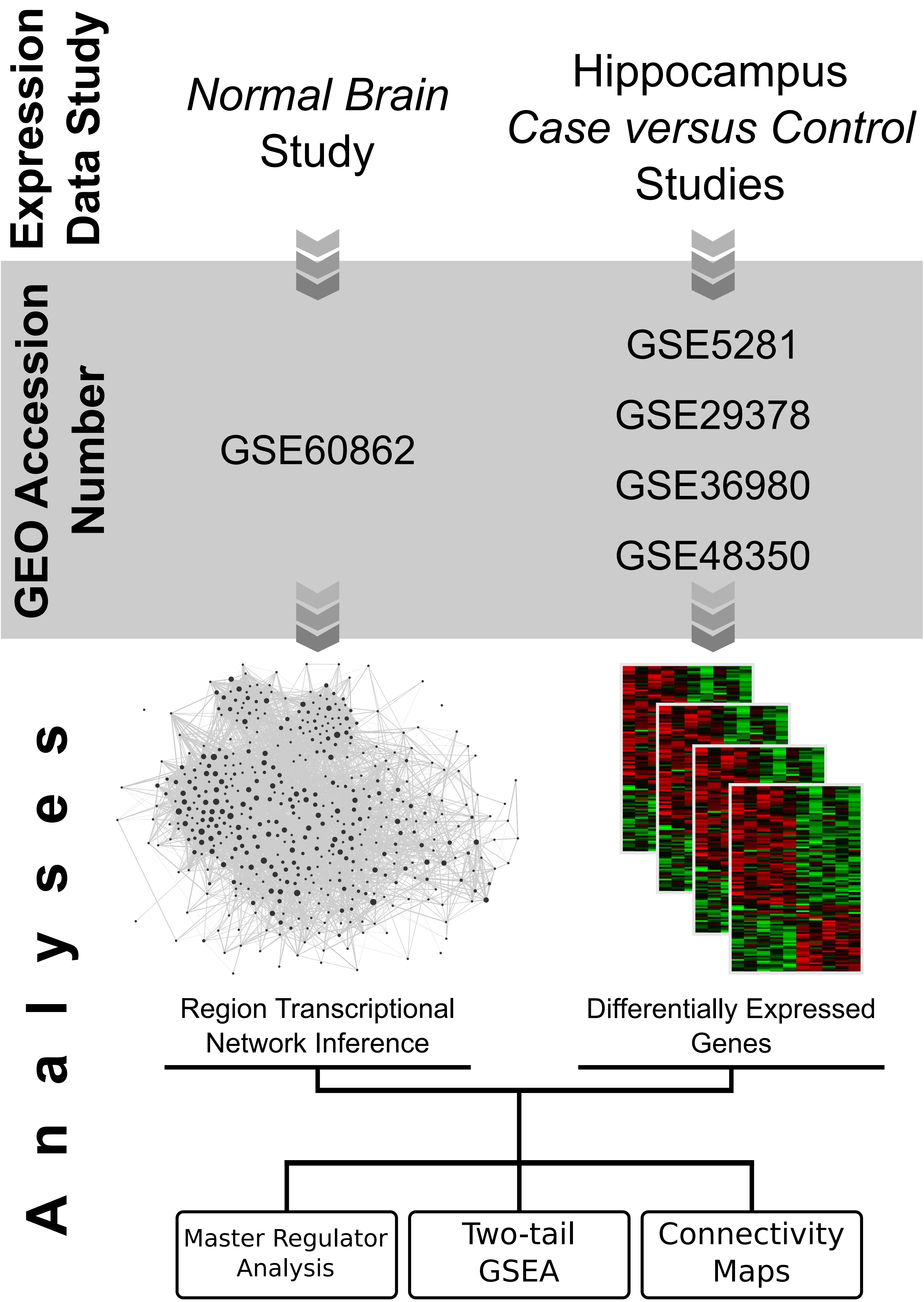
Expression Data Study
Normal Brain Study
Hippocampus Case versus Control Studies
GEO Accession Number
GSE60862
GSE5281
GSE29378
GSE36980
GSE48350
Analyses
Region Transcriptional Network Inference
Differentially Expressed Genes
Master Regulator Analysis
Two-tail GSEA
Connectivity Maps
