# Supplementary figures and images for: Alzheimer’s disease master regulators analysis: search for potential molecular targets and drug repositioning candidates
Source: Alzheimers Res Ther. 2018 Jun 23;10:59. doi: 10.1186/s13195-018-0394-7 (PMC6015462; doi:10.1186/s13195-018-0394-7)

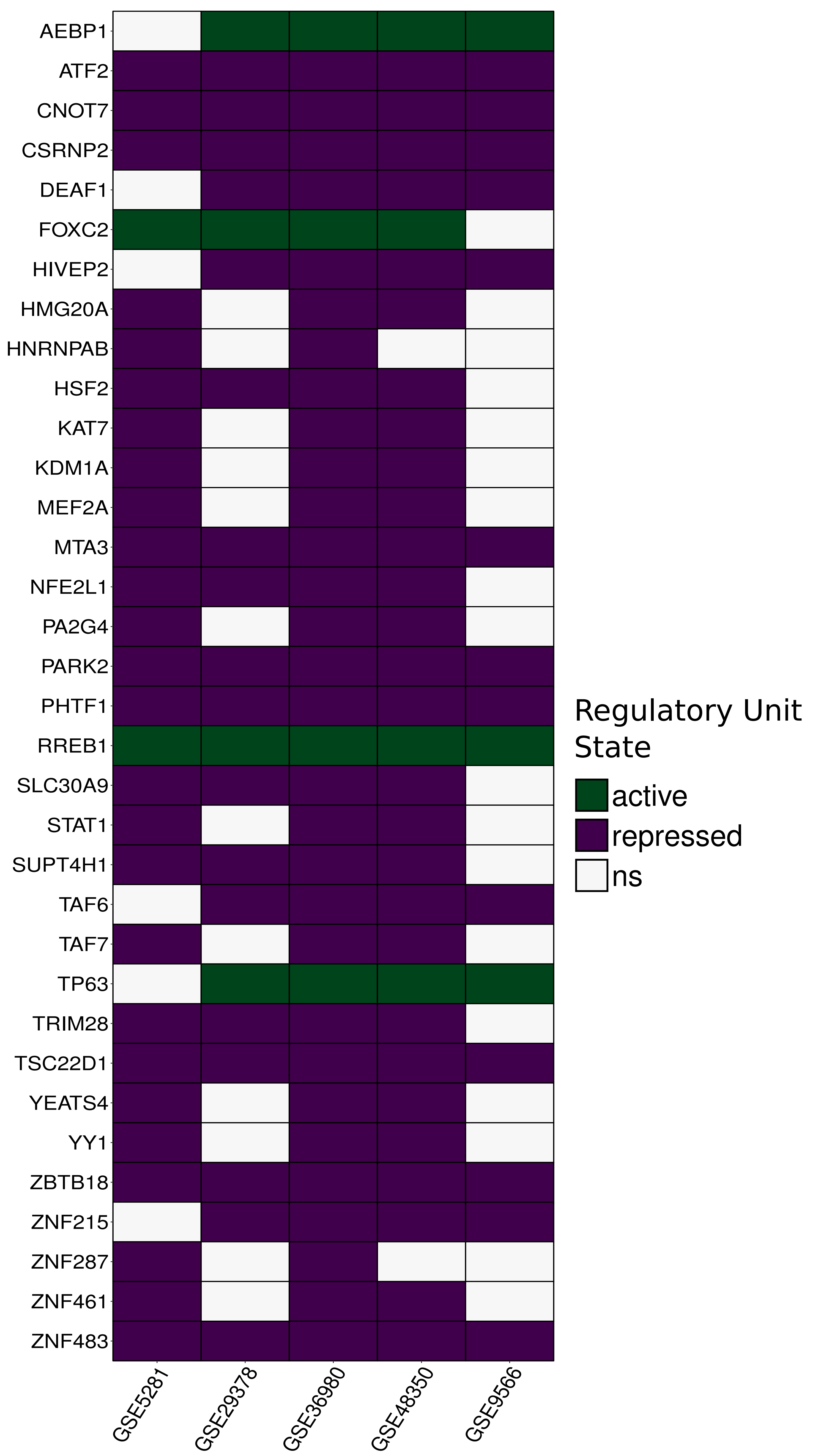

Supplement: Supplementary file 6 — Figure S2. Activation state of MR candidates in AD case-control studies and mouse neuron versus astrocyte data. Tile plot representing the MR candidate state of activation (two-tail gene set enrichment analysis) for the AD case-control (GSE5281, GSE29378, GSE36980, and GSE48350) and mouse neuron versus astrocyte (GSE9566) expression datasets. (PDF 24 kb) [file 13195_2018_394_MOESM6_ESM.pdf]
